# A derivative of PD156707 selectively inhibits NLRP3 inflammasome activation by directly binding to NLRP3

**DOI:** 10.1038/s41598-026-49619-4

**Published:** 2026-04-21

**Authors:** Ye-Rin Jung, Xiang Fei, Hyeong-Min Lee, Anamul Hasan, Eun-Ji Kim, Kyu Tae Byun, Seung-Yong Seo, Tae-Bong Kang, Kwang-Ho Lee

**Affiliations:** 1 Department of Applied Life Science, BK21 Program, Graduate School, Konkuk University, Chungju, 27478 Republic of Korea; 2 Department of Biotechnology, Research Institute (RIBHS), College of Biomedical and Health Science, Konkuk University, Chungju, 27478 Republic of Korea; 3 College of Pharmacy, Gachon University, Incheon, 21936 Republic of Korea; 4 Present Address: Elimland Co., Ltd, Namyangju-si, Gyeonggi-do 12106 Republic of Korea

**Keywords:** NLRP3 inflammasome, Small-molecule inhibitor, DPD (PD-156707 derivative), Biochemistry, Computational biology and bioinformatics, Drug discovery

## Abstract

The NLRP3 inflammasome is a central driver of inflammatory pathology, yet clinical translation of small-molecule inhibitors remains limited by selectivity and safety. Through a 3000-compound screen, we identified PD-156707 as an apparent inhibitor of NLRP3 signaling; however, activity was not reproduced with freshly purchased material. Analytical profiling of the archived sample revealed a previously unrecognized storage-derived derivative, 3-(4-methoxyphenyl)-1-(3,4-methylenedioxyphenyl)-2-(3,4,5-trimethoxybenzylidene)propane-1,3-dione (DPD, 1). DPD, synthesized in a two-step sequence and confirmed to be identical to the degradation product derived from PD-156707, selectively suppressed NLRP3 inflammasome activation in LPS-primed macrophages stimulated with nigericin, ATP, silica, or imiquimod, while sparing AIM2 and NLRC4 pathways. DPD reduced caspase-1 processing and downstream maturation of IL-1β and GSDMD, and impeded ASC oligomerization and speck formation without affecting ASC self-oligomerization in an overexpression system. A DARTS assay indicated direct target engagement of NLRP3, whereas caspase-1 protection was not observed. In silico docking positioned DPD in the NACHT domain (PDB: 7ALV) with a predicted binding affinity of − 9.2 kcal/mol—comparable to MCC950 (– 9.5 kcal/mol)—and suggested interactions with Arg351 within the ATP-binding region. In vivo, DPD reduced IL-1β—but not IL-6—in LPS-induced sepsis and lowered peritoneal IL-1β in MSU-induced peritonitis. Collectively, these data identify DPD as a selective, direct-binding modulator of NLRP3 that disrupts ATP-dependent conformational activation, establishing DPD as a promising lead for inflammasome-targeted therapeutics.

**Supplementary Information:**

The online version contains supplementary material available at 10.1038/s41598-026-49619-4.

## Introduction

Inflammasomes are cytosolic multiprotein complexes that play a critical role in innate immunity by detecting pathogenic or stress-related signals and activating caspase-1, leading to the release of pro-inflammatory cytokines such as IL-1β and IL-18^1,2^. Among them, the NLRP3 inflammasome is the most extensively studied and is implicated in a broad spectrum of inflammatory and metabolic disorders, including gout, type 2 diabetes, atherosclerosis, and neurodegenerative diseases such as Alzheimer’s and Parkinson’s^3–7^.

Given its central role in inflammation, the NLRP3 inflammasome has emerged as a promising therapeutic target^8,9^. Several small-molecule inhibitors, such as MCC950, CY-09, and OLT1177, have demonstrated preclinical efficacy^10–12^. However, clinical translation remains challenging due to limitations in selectivity, bioavailability, and safety^13^. These challenges underscore the need for alternative strategies to identify potent and selective inflammasome inhibitors.

Drug repositioning—the discovery of new therapeutic uses for existing compounds—offers an efficient approach to accelerate the development of inflammasome-targeted agents^14,15^. Repositioned compounds often have established safety and pharmacokinetic profiles, making them attractive candidates for rapid translation to clinical application.

In pursuit of such candidates, our laboratory conducted a chemical library screening to identify small molecules capable of modulating inflammasome activation. During this process, we encountered an unexpected case in which a compound exhibited strong activity that was not reproduced in resynthesized material, prompting further investigation into its chemical stability. This observation ultimately led us to explore a previously unrecognized derivative with potent biological activity.

In this study, we characterize this novel derivative and elucidate its inhibitory mechanism on NLRP3 inflammasome activation, providing insight into its potential as a lead structure for drug repositioning and inflammasome-targeted therapeutics.

## Results

### Identification and validation of a candidate compound inhibiting inflammasome activation

To identify small molecules capable of modulating NLRP3 inflammasome activation, we screened a chemical library of approximately 3000 compounds obtained from the Korea Chemical Bank (Daejeon, Korea). The library included 1280 FDA-approved drugs and 1805 clinical-stage investigational or withdrawn compounds. The screening was performed using a cell-based NLRP3 inflammasome activation assay in which inhibition of nigericin-induced IL-1β secretion served as the primary readout. Briefly, LPS-primed THP-1 cells were treated with library compounds prior to nigericin stimulation, and IL-1β levels in the culture supernatants were quantified by ELISA. Compounds that significantly reduced IL-1β release without affecting cell viability were selected as primary hits. This screening strategy was performed as previously described in our laboratory’s study on inflammasome modulators^16^. Among the screened compounds, one molecule—designated PD-156707—significantly inhibited IL-1β release without affecting cell viability and was therefore selected as a primary hit. However, when the same compound was purchased and retested under identical conditions, the inhibitory activity was not reproduced. This unexpected loss of activity suggested that the original library sample might have undergone chemical modification or degradation during storage, leading us to further examine the integrity of the compound.

**Fig. 1:**
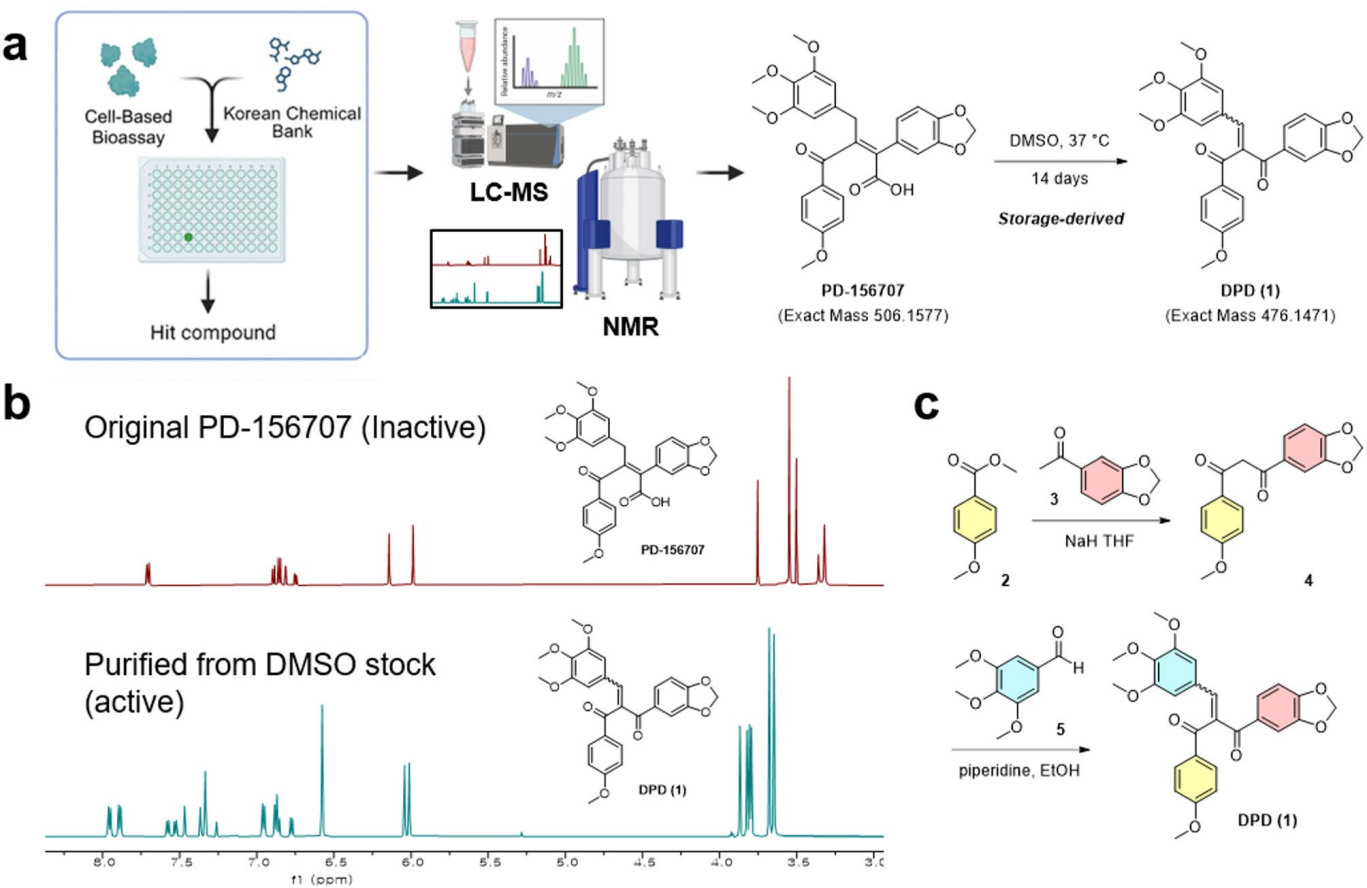
Identification and characterization of DPD as the NLRP3 modulator derived from PD-156707 degradation. (**a**) Screening workflow for NLRP3 inhibitors from the Korean Chemical Bank. Compounds were evaluated based on their ability to inhibit nigericin-induced IL-1β secretion in human macrophage cell line (THP-1). The initial hit PD-156707 was found to undergo a DMSO-mediated conversion at 37 °C for 14 days, providing DPD (1), which retained NLRP3 inhibitory activity. LC-MS analysis revealed the mass shift from 506.1577 to 476.1471. (**b**) ^1^H NMR comparison of PD-156,707 and DPD (1) purified from the degraded DMSO stock. (**c**) Two-step synthesis of DPD (1) for structural and biological studies.

Analytical characterization of the archived PD-156,707 sample by liquid chromatography–mass spectrometry (LC–MS) and nuclear magnetic resonance (NMR) spectroscopy revealed the formation of a degradation-derived compound, designated DPD, responsible for the original screening activity (Fig. 1a,b). The active fraction isolated from the degraded DMSO stock was subjected to structural analysis, and comparative NMR spectroscopy confirmed that its spectrum was identical to that of DPD obtained in a two-step sequence (Fig. 1c). These results confirmed the structure of DPD and clearly demonstrated that the degradation product, not the parent compound PD-156707, is the true NLRP3 modulator.

### DPD selectively inhibits NLRP3 inflammasome activation and downstream caspase-1 signaling

Following the identification of the degradation-derived derivative DPD, we synthesized the compound de novo and evaluated its biological activity in macrophages. LPS-primed bone marrow-derived macrophages (BMDMs) were stimulated with various inflammasome activators—nigericin, ATP, silica, or imiquimod for NLRP3; poly (dA: dT) for AIM2; and flagellin for NLRC4^15,17,18^.

**Fig. 2:**
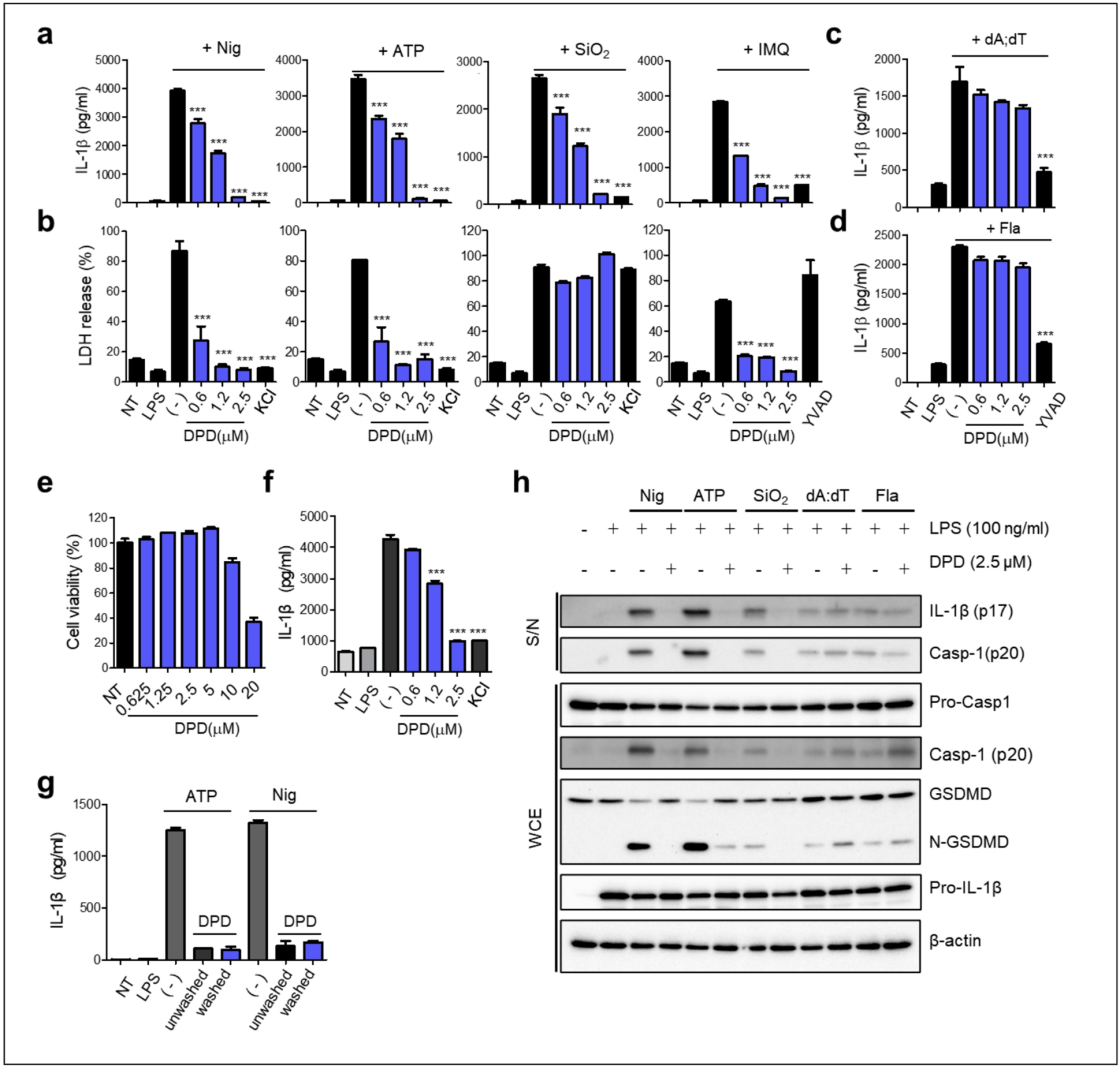
DPD selectively inhibits NLRP3 inflammasome activation and downstream caspase-1 signaling. (**a**,**d**) LPS (100 ng/ml)-primed bone marrow–derived macrophages (BMDMs) were stimulated with inflammasome activators—nigericin (Nig; 10 µM), ATP (5 mM), silica (SiO_2_; 150 µg/ml), or imiquimod (IMQ; 30 µg/ml) for NLRP3; poly(dA: dT) (dA: dT; 1 µg/ml) for AIM2; and flagellin (Fla; 1 µg/ml) for NLRC4—in the presence of the indicated concentrations of DPD. IL-1β secretion was quantified by ELISA. (**b**) Lactate dehydrogenase (LDH) release was measured to assess pyroptotic cell death under the same conditions. (**e**) Cell viability following DPD treatment (up to 20 µM) was evaluated by MTT assay. (**f**) Differentiated human THP-1 macrophage-like cells were primed with 100 ng/ml of LPS and stimulated with nigericin (10 µM) in the presence or absence of DPD, and the release of IL-1β was measured by ELISA. (**g**) Wash-out experiments were performed in which DPD was removed prior to inflammasome activation, followed by stimulation with NLRP3 activators, and IL-1β secretion was measured to assess the persistence of DPD-mediated inhibition. (**h**) Immunoblot analysis of caspase-1, IL-1β, and gasdermin D (GSDMD) cleavage in LPS-primed BMDMs stimulated with the indicated inflammasome activators in the presence or absence of DPD (2.5 µM). Whole cell lysates (WCE) and supernatants (S/N) were analyzed by immunoblotting.***P < 0.001 vs. respective stimulated control.

Under these conditions, DPD treatment dose-dependently reduced IL-1β secretion and lactate dehydrogenase (LDH) release in cells stimulated with NLRP3 activators (Fig. 2a,b), whereas it had minimal effects on AIM2- or NLRC4-mediated responses (Fig. 2c,d). Notably, in the case of SiO_2_ stimulation, DPD effectively suppressed IL-1β secretion but did not attenuate LDH release. This discrepancy may reflect the particulate nature of SiO_2_, which can induce lysosomal damage and inflammasome-independent membrane disruption^19,20^. In addition, imiquimod (IMQ)-induced LDH release was only modestly affected by the caspase-1 inhibitor YVAD (Fig. 2a), consistent with reports that inflammasome activation can trigger cell death even when caspase-1 activity is inhibited^21^.

Importantly, DPD exhibited no detectable cytotoxicity at concentrations up to 5 µM, as determined by MTT assay (Fig. 2e), excluding nonspecific cell death as a cause of the observed inhibitory effects.

Given this lack of cytotoxicity, we next examined whether the inhibitory activity of DPD was reproducible in human cells. Accordingly, differentiated THP-1 macrophage-like cells were stimulated with nigericin, and DPD similarly attenuated NLRP3 inflammasome-associated responses (Fig. 2f), supporting the translational relevance of DPD. In addition to its conserved activity across species, wash-out experiments revealed that removal of DPD prior to inflammasome activation failed to restore IL-1β secretion, demonstrating that DPD-mediated inhibition of NLRP3 inflammasome activation persists even after compound removal (Fig. 2g). These findings suggest that DPD induces a durable suppression of inflammasome responsiveness rather than acting as a transient inhibitor.

To elucidate the molecular basis of this sustained inhibition, caspase-1 activation and substrate cleavage were examined by immunoblotting. Consistent with the functional data, DPD markedly suppressed the processing of pro-caspase-1 (p45) to its active p20 form and concomitantly reduced IL-1β maturation (p17) and gasdermin D cleavage (GSDMD-N) in macrophages stimulated with NLRP3 activators (Fig. 2h). In contrast, DPD had no detectable effect on caspase-1 activation or downstream substrate processing in AIM2- or NLRC4-stimulated cells^22–24^.

Collectively, these results demonstrate that DPD selectively inhibits NLRP3 inflammasome activation and downstream caspase-1-dependent pyroptotic signaling without affecting cell viability or other inflammasome pathways.

### DPD blocks ASC oligomerization and speck formation

**Fig. 3:**
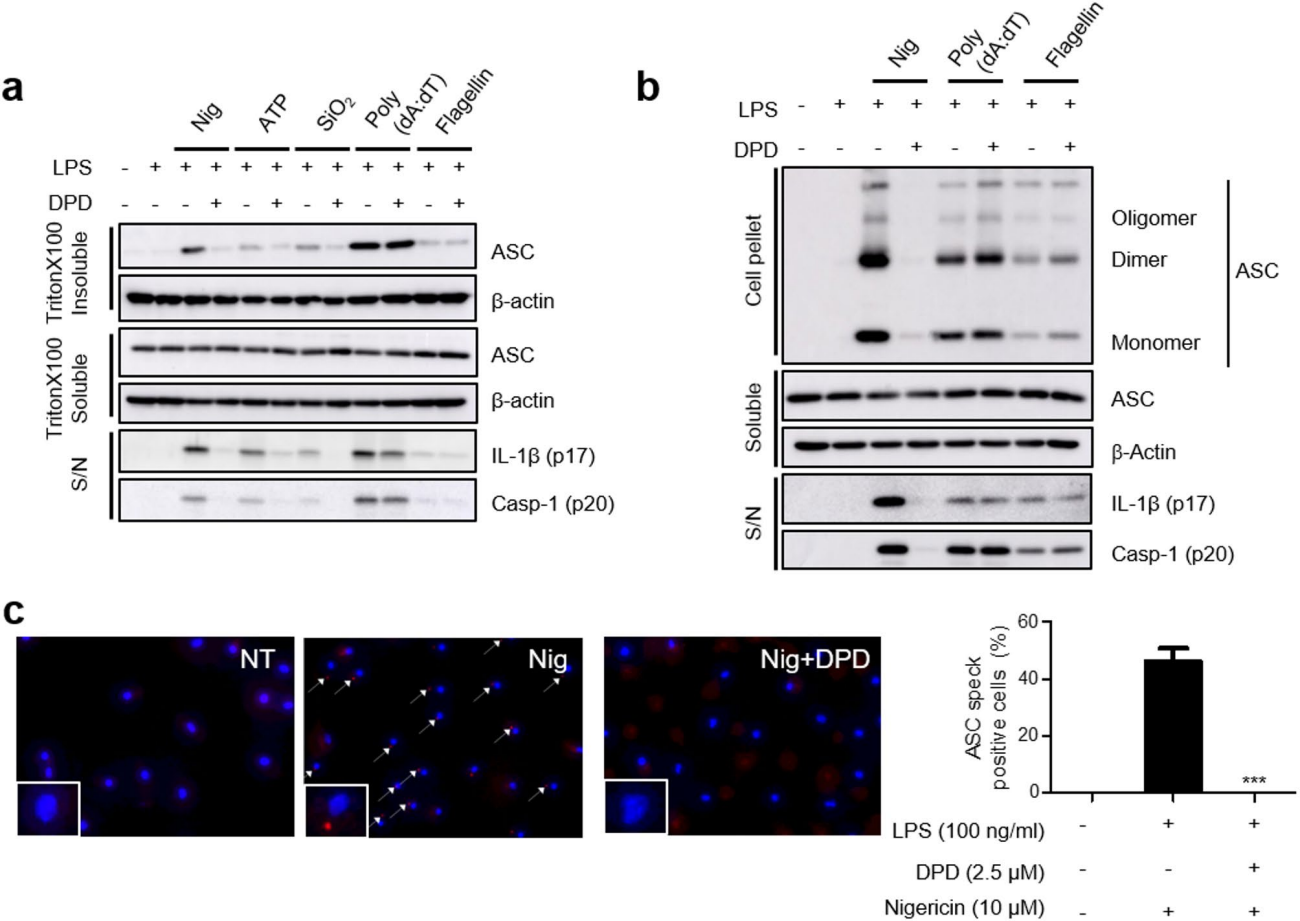
DPD blocks ASC oligomerization and speck formation in LPS-primed macrophages. (**a**) Fractionation analysis of ASC distribution between Triton X-100—soluble and—insoluble fractions in BMDMs stimulated with NLRP3, AIM2, or NLRC4 activators in the presence or absence of 2.5 µM DPD. (**b**) DSS cross-linking assay examining ASC oligomerization in nigericin-stimulated BMDMs. (**c**) Immunofluorescence images showing ASC speck formation in nigericin-stimulated macrophages treated with or without DPD. Quantification of ASC specks per cell using ImageJ. Data are presented as mean ± SEM (*n* = 3). ***P < 0.001 vs. nigericin-stimulated group.

Since DPD inhibited caspase-1 activation in Fig. 2, we next examined its effects on upstream events required for inflammasome assembly. Caspase-1 activation depends on the oligomerization of ASC, which aggregates into perinuclear specks upon recruitment by NLRP3^25,26^. LPS-primed BMDMs were treated with DPD and stimulated with various inflammasome activators (nigericin, ATP, silica, poly (dA: dT), or flagellin). Fractionation analysis showed that DPD markedly inhibited the translocation of ASC to the Triton X-100–insoluble fraction following NLRP3 activation but had no effect on AIM2 or NLRC4-mediated ASC redistribution (Fig. 3a). Consistently, DSS cross-linking and immunocytochemistry demonstrated that DPD suppressed ASC oligomerization and speck formation in nigericin-stimulated macrophages, without affecting poly (dA: dT)- or flagellin-induced ASC assembly (Fig. 3b,c).

To determine whether DPD directly targets ASC, HEK293T cells overexpressing EGFP-tagged ASC were treated with DPD at different time points relative to transfection. DPD was added either 4 h before transfection, at the time of transfection, or 4 h after transfection. Upon all treatment conditions, DPD did not suppress ASC speck formation in this overexpression system (Supplementary Fig. 1), indicating that DPD does not directly inhibit ASC but rather interferes with upstream events leading to its oligomerization.

### DPD directly interacts with NLRP3 to inhibit its activation

**Fig. 4:**
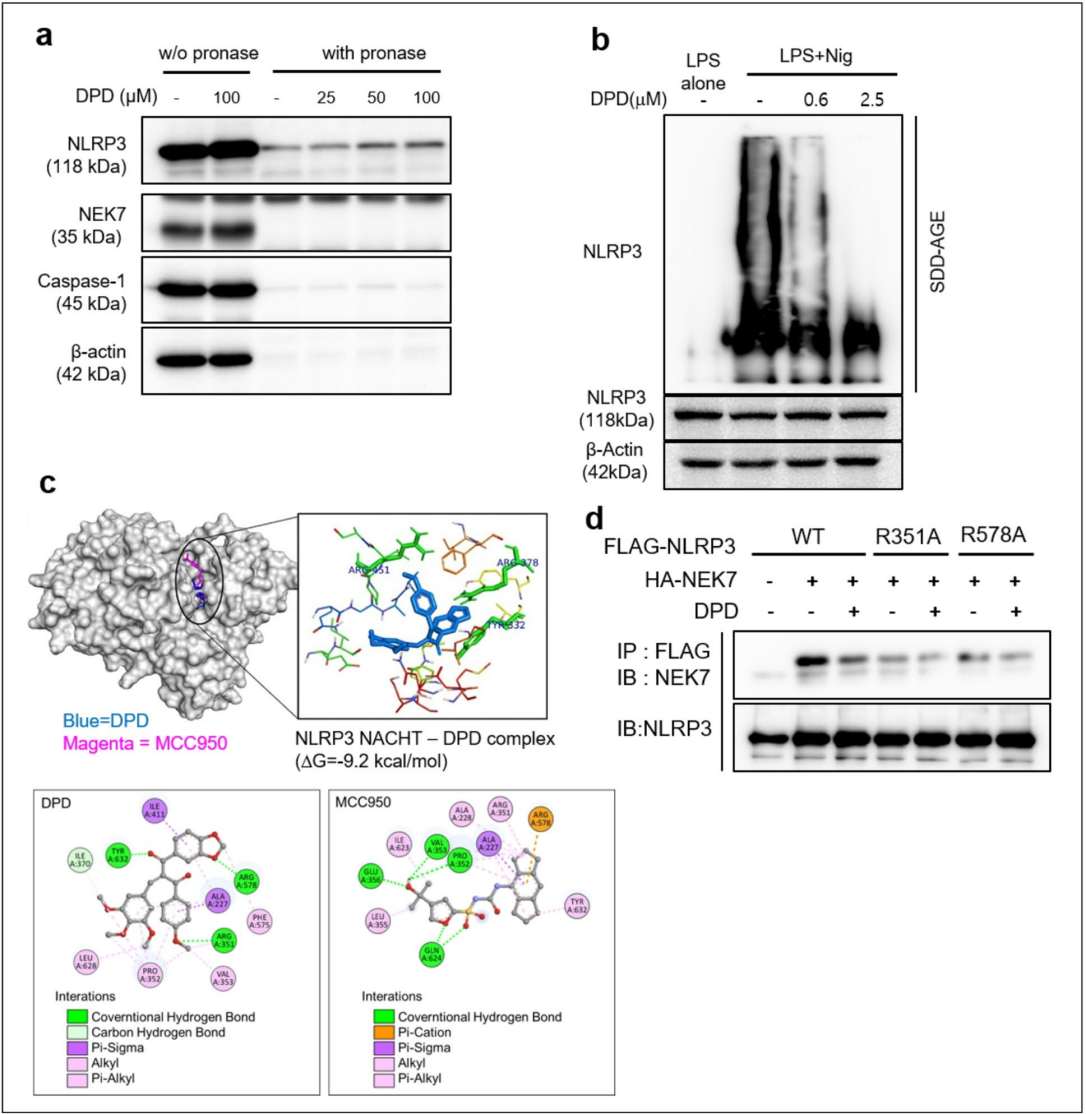
DPD directly binds NLRP3 and disrupts its oligomerization. (**a**) Drug affinity responsive target stability (DARTS) assay was performed to examine the direct binding of DPD to NLRP3 in cell lysates. LPS-primed J774A.1 cells were lysed using TTNE lysis buffer and incubated with DMSO or the indicated concentrations of DPD for 30 min. The lysates were then digested with pronase at a 1:400 pronase-to-protein ratio for an additional 30 min, followed by immunoblot analysis to assess protease resistance of NLRP3. (**b**) DPD suppresses NLRP3 oligomerization as analyzed by semi-denaturing detergent agarose gel electrophoresis (SDD-AGE). LPS-primed bone marrow–derived macrophages (BMDMs) were pretreated with 2.5 µM DPD for 1 h and subsequently stimulated with nigericin (10 µM) for 30 min. Insoluble protein fractions were isolated and subjected to SDD-AGE to detect oligomerized NLRP3 by immunoblotting using an anti-NLRP3 antibody (upper), whereas the soluble fractions were separated by SDS–PAGE and analyzed in parallel (lower). (**c**) The upper panel shows molecular docking model showing the predicted binding pose of DPD within the NACHT domain of NLRP3 whereas the lower panel shows two-dimensional interaction diagrams illustrating the predicted molecular interactions between the NACHT domain and DPD (left) or MCC950 (right). (**d**) Interaction between NLRP3 and NEK7 analyzed by co-immunoprecipitation. HEK293T cells were transfected with HA-tagged NEK7 together with FLAG-tagged wild-type (WT) or mutant NLRP3 (R351A or R578A) in the presence or absence of DPD (2.5 µM). Cell lysates were subjected to immunoprecipitation using an anti-FLAG antibody, and the association of NEK7 with NLRP3 was analyzed by immunoblotting with an anti-NEK7 antibody.

As shown in Fig. 2, DPD also inhibited IL-1β secretion in cells stimulated with imiquimod, a K^+^ efflux-independent NLRP3 activator, suggesting that its inhibitory effect is unlikely to arise from blocking upstream ionic fluxes^27^. To explore whether this inhibition involves a direct interaction with NLRP3 inflammasome components, we performed a drug affinity responsive target stability (DARTS) assay. The assay showed that DPD dose-dependently protected NLRP3, but not NEK7, caspase-1, or β-actin, from proteolytic degradation (Fig. 4a), indicating a direct physical interaction between DPD and NLRP3^28–30^. Consistent with this interaction, we next examined whether DPD affects NLRP3 oligomerization, a critical step in inflammasome assembly. Semi-denaturing detergent agarose gel electrophoresis (SDD-AGE) revealed that DPD markedly suppressed nigericin-induced NLRP3 oligomerization in LPS-primed macrophages (Fig. 4b), suggesting that DPD interferes with the higher-order assembly of NLRP3 required for inflammasome activation.

To further investigate the structural basis of this interaction, we performed in silico molecular docking using AutoDock Vina^31^. The crystal structure of the human NLRP3 NACHT domain (PDB ID: 7ALV) was used as the receptor, and the 3D structure of DPD was modeled and energy-minimized prior to docking^32–34^. The simulation demonstrated that DPD binds stably within the ATP-binding pocket of the NACHT domain, a region known to regulate ATPase activity and oligomerization, with a predicted binding affinity of − 9.2 kcal/mol—comparable to the well-characterized NLRP3 inhibitor MCC950 (– 9.5 kcal/mol) (Fig. 4b). DPD formed conventional hydrogen bonds with Arg351 (2.77 A°), Arg578 (2.71 A°), and Tyr632 (2.08 A°), as well as a carbon hydrogen bond with Ile370 (3.60 A°). Additional hydrophobic contacts, including π–σ interactions with Ala227 (3.73 A°) and Ile411 (3.54 A°), and alkyl or π-alkyl contacts with Arg351, Pro352, Val353, Leu628, and Phe575, contributed to complex stability (Fig. 4c; Table 1). Notably, both DPD and MCC950 interacted with Arg351, a γ-phosphate-sensing residue within the Sensor-1 motif critical for inter-domain organization of the NACHT domain.

To experimentally validate the residues predicted to interact with DPD, we generated NLRP3 mutants targeting Arg351 and Arg578 and examined their effects in a reconstituted inflammasome system in HEK293T cells. While DPD markedly suppressed inflammasome activation in cells expressing wild-type NLRP3, this inhibitory effect was substantially reduced in cells expressing the R351A or R578A mutants (Fig. 4d), supporting the docking prediction that these residues contribute to the interaction between DPD and NLRP3.

Together, these results support the conclusion that DPD directly interacts with NLRP3 and likely interferes with its ATP-dependent conformational activation, thereby preventing inflammasome assembly.

**Table 1:** Predicted non-bonded interactions between the NACHT domain of NLRP3 and DPD or MCC950.

Residues	Distances	Category	Type
DPD (− 9.2 kcal/mol)			
ARG351	2.77	Hydrogen bond	Conventional hydrogen bond
ARG578	2.71	Hydrogen bond	Conventional hydrogen bond
TYR632	2.08	Hydrogen bond	Conventional hydrogen bond
ILE370	3.60	Hydrogen bond	Carbon hydrogen bond
ALA227	3.73	Hydrophobic	Pi-Sigma
ILE411	3.54	Hydrophobic	Pi-Sigma
ARG351	3.59	Hydrophobic	Alkyl
PRO352	3.97	Hydrophobic	Alkyl
VAL353	3.38	Hydrophobic	Alkyl
LEU628	5.07	Hydrophobic	Alkyl
PRO352	4.16	Hydrophobic	Alkyl
PHE575	5.44	Hydrophobic	Pi-Alkyl
ALA227	4.51	Hydrophobic	Pi-Alkyl
MCC950 (− 9.5 kcal/mol)			
GLU356	2.26	Hydrogen bond	Conventional hydrogen bond
GLN624	2.35	Hydrogen bond	Conventional hydrogen bond
GLN624	2.70	Hydrogen bond	Conventional hydrogen bond
PRO352	2.62	Hydrogen bond	Conventional hydrogen bond
VAL353	2.10	Hydrogen bond	Conventional hydrogen bond
ARG578	4.47	Electrostatic	Pi-Cation
ALA227	3.76	Hydrophobic	Pi-Sigma
ALA228	5.30	Hydrophobic	Alkyl
ARG351	4.69	Hydrophobic	Alkyl
PRO352	4.46	Hydrophobic	Alkyl
VAL353	4.55	Hydrophobic	Alkyl
LEU355	4.59	Hydrophobic	Alkyl
ILE623	4.37	Hydrophobic	Alkyl
TYR632	4.77	Hydrophobic	Pi-Alkyl
PRO352	5.11	Hydrophobic	Pi-Alkyl

### DPD alleviates NLRP3-dependent inflammation in vivo

To evaluate whether the NLRP3 inhibitory activity of DPD observed in vitro translates to in vivo anti-inflammatory efficacy, we examined its effects in LPS-induced sepsis and MSU-induced peritonitis mouse models^35,36^.

**Fig. 5:**
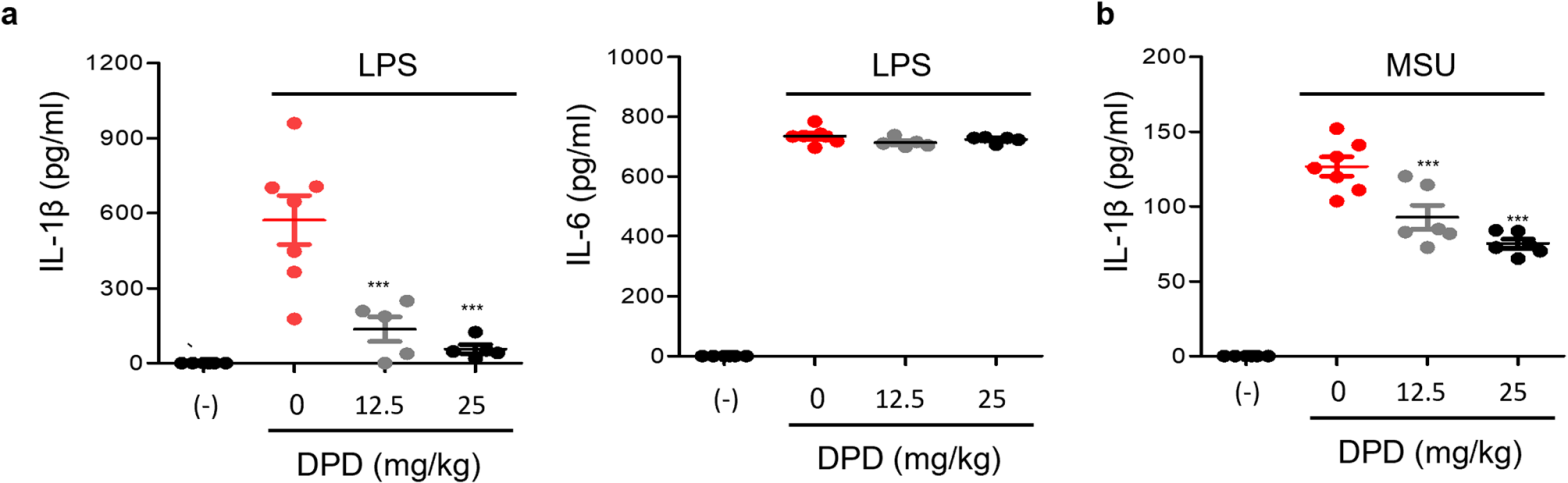
DPD suppresses IL-1β production in mouse models of inflammation. (**a**) Serum IL-1β and IL-6 levels measured by ELISA in LPS (20 mg/kg)-induced sepsis model mice treated with or without indicated amount of DPD. (**b**) Peritoneal IL-1β levels in MSU (50 mg/kg)-induced peritonitis model following DPD administration. Data are shown as mean ± SEM (*n* = 5 per group). Statistical analysis by one-way ANOVA with Tukey’s post hoc test; ***P <  0.001 compared with the LPS injection alone group.

In the LPS-induced sepsis model, intraperitoneal injection of LPS elicited a robust systemic inflammatory response involving both canonical and non-canonical inflammasome pathways. Notably, LPS-induced inflammation is primarily initiated through caspase-11 dependent non-canonical inflammasome signaling, which can subsequently promote NLRP3 inflammasome activation through gasdermin D-mediated membrane permeabilization and potassium efflux^37–39^. Under these conditions, administration of DPD (10 and 25 mg/kg) significantly reduced serum IL-1β levels in a dose-dependent manner, indicating suppression of inflammasome-associated cytokine production in vivo. In contrast, serum IL-6 levels, which are largely regulated independently of inflammasome activation, were not affected by DPD treatment (Fig. 5a). These data suggest that DPD preferentially attenuates inflammasome-related inflammatory outputs rather than broadly suppressing systemic inflammation.

To further examine inflammasome involvement under conditions more directly linked to NLRP3 activation, we employed the MSU-induced peritonitis model, a well-established NLRP3-dependent inflammatory setting. Consistent with the in vitro findings, DPD treatment markedly reduced IL-1β levels in peritoneal lavage fluid in a dose-dependent manner following MSU challenge (Fig. 5b).

Collectively, these results demonstrate that DPD suppresses inflammasome-associated IL-1β production in vivo. While multiple inflammasome pathways may contribute to inflammatory responses in the LPS-induced sepsis model, the consistent inhibitory effect of DPD in the MSU peritonitis model supports the conclusion that its in vivo anti-inflammatory activity is closely associated with modulation of NLRP3 inflammasome signaling.

## Discussion

In this study, we identify DPD as a selective small-molecule inhibitor of the NLRP3 inflammasome. DPD emerged unexpectedly as a storage-derived derivative of the screening hit PD-156707, yet it reproducibly suppressed NLRP3-dependent IL-1β release, caspase-1 activation, and GSDMD cleavage in macrophages without overt cytotoxicity. DPD showed minimal effects on AIM2 or NLRC4 inflammasomes, indicating target selectivity within the inflammasome family. Importantly, in vivo administration of DPD reduced IL-1β levels in both LPS-induced sepsis and MSU-induced peritonitis models while leaving IL-6 unchanged, supporting on-pathway activity rather than broad immunosuppression.

Biochemical and cell biological assays converge on a mechanism in which DPD acts upstream of ASC oligomerization yet downstream of common proximal triggers such as K^+^ efflux. DARTS assays demonstrated direct engagement of NLRP3, and in silico docking positioned DPD within the NACHT domain—a region governing ATPase activity and oligomerization—with a predicted binding energy of − 9.2 kcal/mol. Docking analysis suggested interactions with Arg351 and Arg578 within the ATP-binding region. Notably, Arg351 resides in the Sensor-1 motif that has been hypothesized to sense the γ-phosphate of ATP and contribute to domain interface organization^40,41^. To experimentally test this structural prediction, we introduced point mutations at these residues in a reconstituted inflammasome system. Whereas DPD efficiently suppressed inflammasome activation in cells expressing wild-type NLRP3, its inhibitory effect was markedly attenuated in cells expressing the R351A or R578A mutants. These results provide functional support for the docking model and indicate that these residues contribute to the interaction between DPD and the NLRP3 NACHT domain. Consistent with interference at this module, DPD suppressed NLRP3 oligomerization as detected by SDD-AGE and curtailed ASC speck formation and insoluble fraction translocation upon NLRP3 activation. In contrast, DPD did not inhibit ASC self-oligomerization in an overexpression system, indicating that DPD does not directly target ASC. Together, these data support a model in which DPD binds NLRP3 to impede ATP-dependent conformational transitions required for oligomerization and inflammasome assembly.

The predicted affinity of DPD for the NACHT domain is comparable to MCC950 (– 9.5 kcal/mol in our docking pipeline), a benchmark NLRP3 inhibitor. Functionally, DPD phenocopies MCC950 in suppressing NLRP3-dependent caspase-1 activation and IL-1β maturation. However, its origin from PD-156707, a previously reported endothelin receptor antagonist developed for cardiovascular disorders, distinguishes it from de novo chemical matter. The conversion of PD-156707 into DPD during storage unexpectedly yielded a compound with enhanced NLRP3 inhibitory activity, suggesting that structural modification of known chemical scaffolds can generate previously unrecognized biological activities.

Because DPD arises from a storage-associated conversion of PD-156707, it is possible that trace amounts of DPD could have been present in PD-15607 preparations used in previous studies, particularly under conditions that favor chemical instability. Under such conditions, DPD may have contributed to some pharmacological effects previously attributed to PD-156707. However, most prior investigations of PD-156707 focused on endothelin receptor signaling and did not examine inflammasome-dependent pathways.

DPD selectively restrained NLRP3 outputs across diverse stimuli (nigericin, ATP, silica and imiquimod), including K^+^-efflux–independent activation (imiquimod), strengthening the view that DPD targets NLRP3 itself rather than ion fluxes or generic stress pathways. Lack of inhibition in AIM2 and NLRC4 contexts and the absence of IL-6 reduction in vivo further support on-target activity. These features are desirable for anti-inflammatory therapeutics aiming to preserve host defense mechanisms outside the NLRP3 axis.

The in vivo efficacy of DPD in two mechanistically complementary models (systemic endotoxemia and crystal-induced peritonitis) suggests that DPD can access relevant compartments and engage the target in physiological settings. Because excessive NLRP3 activity underlies a spectrum of metabolic, sterile inflammatory, and neuroinflammatory disorders, DPD provides a lead scaffold for indications in which MCC950-like pharmacology has shown benefit preclinically.

There are some limitations to this study. Although mutational analysis supports the predicted interaction between DPD and the NACHT domain, high-resolution structural evidence-such as cryo-EM or co-crystallization will be necessary to definitively define the binding mode more precisely. In addition, broader profiling will be required to exclude potential off-target effects and to determine pharmacokinetic properties, metabolic stability, and long-term safety in vivo. Finally, because DPD is derived from PD-156707 through a storage-associated chemical transformation, further investigation will be needed to determine its stability and suitability for pharmaceutical formation.

Mechanistically, our data indicate that DPD interferes with the NEK7-NLRP3 interaction, likely through binding to the NACHT domain. Future studies will be required to determine how this interaction affects the stepwise assembly and activation of the NLRP3 inflammasome complex.

In summary, DPD emerges as a selective, direct-binding antagonist of NLRP3 that suppresses inflammasome activation by targeting the NACHT domain. Its efficacy across cellular and animal models, together with its mechanistically supported interaction with key residues in the NACHT ATP-binding region, positions DPD as a promising lead compound for NLRP3-targeted therapy and provides insight into how structural modification of existing chemical scaffolds can yield modulators of innate immune signaling pathways.

## Methods

### Reagents

Lipopolysaccharide (LPS, L2630), adenosine triphosphate (ATP, A7699), Ac-YVAD-fmk (caspase-1 inhibitor), β-mercaptoethanol (β-ME), MTT reagent (3-(4,5-dimethylthiazol-2-yl)-2,5-diphenyltetrazolium bromide), DMSO, DAPI, and Cy3-conjugated goat anti-mouse IgG were purchased from Sigma-Aldrich (St. Louis, MO, USA). Nigericin (tlrl-nig), silica (tlrl-sol), imiquimod, poly(dA: dT), flagellin, MSU crystals (tlrl-msu) and mouse IL-6 ELISA kit were obtained from InvivoGen (San Diego, CA, USA). The known NLRP3 inhibitor MCC950 was purchased from Cayman Chemical (Ann Arbor, MI, USA). The LDH Cytotoxicity Detection Kit was obtained from Thermo Fisher Scientific (USA). Lipofectamine 2000 (Invitrogen) and JetPEI (Polyplus-transfection, France) were used for transfection. The Mouse IL-1β/IL-1F2 DuoSet ELISA kit was from R&D Systems (Minneapolis, MN, USA). BCA Protein Assay Kit, ECL detection reagents, DSS (disuccinimidyl suberate), and Tris-HCl buffer were obtained from Thermo Fisher Scientific.

### Chemical synthesis, and analytical characterization

Two-step synthesis of DPD (1). To a stirred suspension of NaH (60% dispersion in mineral oil) in dry THF (4 ml) in a 25 ml round-bottom flask under N2 atmosphere, a solution of methyl 4-methoxybenzoate (2, 242 mg, 1.46 mM) and 3′,4′-(methylenedioxy) acetophenone (3, 200 mg, 1.22 mM) in dry THF was added with stirring and placed in an ice bath. After stirred under reflux for 10 h, The mixture was cooled and filtered through Celite pad. The solid was washed with EtOH (20 ml). The combined filtrates were poured into Et2O (20 ml) and aq. HCl (1 M, 20 ml). The aqueous layer was extracted with Et2O twice. The combined extracts were washed with brine 3 times and dried. Evaporation and purify the residue by flash column chromatography on silica to obtain compound 4 (160 mg, 45%). Then, 1-(1,3-benzodioxol-5-yl)-3-(4-methoxyphenyl)-1,3-propanedione (4, 100 mg, 36 mM) and 3,4,5-trimethoxybenzaldehyde (79 mg, 40 mM) was stirred at ambient temperature for 30 min. Piperidine (0.1 ml, 1.01 mM, 3.0 eq) and glacial acetic acid (0.5 ml) were subsequently added. The reaction mixture was heated to reflux and stirred for 24 h. After cooling to room temperature, the solvent was removed under reduced pressure. The resulting crude residue was purified by flash column chromatography to yield DPD (1, 80 mg, 50%) as a yellow solid. 1 H NMR (600 MHz, CDCl3) δ 7.97 (dt, J = 9.0, 1.9 Hz, 1 H), 7.93–7.88 (m, 1 H), 7.62–7.56 (m, 1 H), 7.51–7.46 (m, 1 H), 7.34 (d, J = 1.4 Hz, 1 H), 7.00–6.94 (m, 1 H), 6.94–6.82 (m, 2 H), 6.59 (s, 2 H), 6.11–6.06 (m, 1 H), 6.03 (d, J = 0.9 Hz, 1 H), 3.92–3.68 (m, 12 H); 13 C{1 H} NMR (150 MHz, CDCl3) δ 195.46, 195.31, 193.25, 192.90, 164.26, 163.37, 153.05, 153.02, 152.63, 151.68, 148.48, 148.09, 141.85, 141.73, 139.71, 139.13, 139.11, 132.12, 131.87, 131.56, 130.08, 129.69, 128.60, 128.57, 126.77, 126.25, 114.21, 113.81, 109.57, 108.48, 108.42, 108.35, 107.92, 107.48, 107.40, 102.04, 101.92, 60.91, 55.98, 55.94, 55.54; HRMS (EI+) calculated for C27H24O8, [M + H] + = 476.1471 found: 476.1468.

### Cell culture

Bone marrow cells were isolated from the femurs and tibias of 6–8-week-old C57BL/6 mice using sterile phosphate-buffered saline (PBS) containing 2% fetal bovine serum (FBS, Gibco). The cells were differentiated into bone marrow-derived macrophages (BMDMs) in RPMI-1640 medium (Gibco) supplemented with 10% FBS, 1% penicillin–streptomycin (P/S), 50 µM β-mercaptoethanol, 1 mM sodium pyruvate, MEM non-essential amino acids, and 30% L929 cell-conditioned medium (LCM) for 7 days. The culture medium was replaced on days 3 and 6, and differentiated cells were harvested on day 7 for experiments. L929 cells were maintained in Dulbecco’s Modified Eagle’s Medium (DMEM) containing 10% FBS and 1% P/S in T75 culture flasks. After 7 days, the culture supernatant was collected, filtered through a 0.22 μm filter, and stored at − 80 °C for future use. J774A.1 and HEK293T cells were cultured in DMEM supplemented with 10% FBS and 1% P/S. All cells were maintained at 37 °C in a humidified incubator with 5% CO_2_.

### Animals

Female C57BL/6 mice (6 weeks old) were purchased from Orient Bio Co. (Seongnam, Korea). Animals were housed in groups of five under standard laboratory conditions (22 ± 2 °C, 55 ± 5% humidity, 12 h light/dark cycle) with free access to food and water. All animal experiments were performed in accordance with the guidelines of the Konkuk University Animal Care and Use Committee. The experimental protocol was reviewed and approved by the Ethics of Animal Experiments Committee of Konkuk University (approval number: KU23228). All animal experiments were performed and reported in accordance with the ARRIVE guidelines.

### Inflammasome activation

For NLRP3 inflammasome activation, BMDMs were primed with 100 ng/ml of LPS for 3 h and washed with warm PBS. Experiments were subsequently performed in Opti-MEM medium. Cells were pretreated with indicated concentration of DPD, KCl (150 mM), or Y-VAD (50 µM) for 1 h, followed by stimulation with 10 µM nigericin or 5 mM ATP for 1 h, and 150 µg/ml silica for 3 h.

To activate AIM2 and NLRC4 inflammasomes, LPS-primed BMDMs were transfected with poly (dA: dT) (1 µg/ml, 2 h) or flagellin (1 µg/ml, 3 h) using Lipofectamine 2000. For K^+^ efflux-independent activation, LPS-primed BMDMs were stimulated with imiquimod (30 µg/ml) for 3 h in the presence or absence of DPD. Supernatants were collected for cytokine analysis by ELISA. To evaluate the reversibility of DPD-mediated NLRP3 inhibition, LPS-primed BMDMs were pretreated with DPD (2.5 µM) for 1 h, washed three times with PBS, and incubated in compound-free Opti-MEM prior to stimulation, while DPD was maintained throughout the experiment in non-wash controls. Cells were then stimulated with nigericin (10 µM) or ATP (5 mM) for 1 h, and inflammasome activation was assessed by IL-1β ELISA.

### Chemical library screening

A small-molecule chemical library was obtained from the Korea Chemical Bank (Daejeon, Korea).

The library comprised approximately 3000 compounds, including 1280 FDA-approved drugs and 1805 investigational or withdrawn clinical-stage compounds (Phase I–III).

All compounds were supplied as 5 mM stock solutions in DMSO and stored at − 20 °C until use.

LPS-primed BMDMs seeded on 96-well plates were stimulated with nigericin (10 µM) for 1 h to induce NLRP3 inflammasome activation. Compounds from the library were added 1 h before nigericin stimulation at a final concentration of 5 µM.

After stimulation, cell-free supernatants were collected and analyzed for IL-1β secretion using ELISA. The relative inhibition rate was calculated by comparing IL-1β levels in compound-treated wells with those of vehicle-treated controls. Compounds showing ≥ 50% reduction in IL-1β release without affecting cell viability were selected as primary hits and further validated in secondary assays.

### Enzyme-linked immunosorbent assay (ELISA)

IL-1β or IL-6 levels in cell supernatants were quantified using a Mouse IL-1β/IL-1F2 DuoSet ELISA kit or mouse IL-6 ELISA kit according to the manufacturer’s instructions. Absorbance was measured at 450 nm using a microplate reader (Multiskan GO, Thermo Fisher Scientific, USA).

### Cell viability and cytotoxicity assays

The effects of DPD on cell viability and cytotoxicity were assessed using MTT and LDH assays, respectively. Bone marrow-derived macrophages (BMDMs; 2.5 × 10^4^ cells/well) were seeded in 96-well plates (SPL, 30096, Korea) and incubated overnight. Cells were treated with various concentrations of DPD for 8 h, followed by incubation with MTT reagent (500 µg/ml) for 2 h. After removal of the medium, formazan crystals were dissolved in DMSO, and absorbance was measured at 550 nm using a microplate reader. For LDH assays, supernatants were collected by centrifugation (6000 rpm, 4 °C, 5 min) after DPD treatment and analyzed using a commercial LDH assay kit according to the manufacturer’s instructions. Absorbance was measured at 450 nm, and LDH release was expressed as a percentage of total cell lysis.

### Protein extraction and immunoblotting

Cells were lysed in RIPA buffer containing protease and phosphatase inhibitor cocktails for 20 min at 4 °C, and lysates were centrifuged at 13,000 rpm for 15 min. Protein concentrations were determined using a BCA assay. Equal amounts of protein were separated by SDS–PAGE and transferred to nitrocellulose membranes. Membranes were blocked with 5% skim milk in PBS containing 0.1% Tween 20 (PBST) for 1 h, incubated with primary antibodies overnight at 4 °C, and then with HRP-conjugated secondary antibodies for 1 h at room temperature. Protein bands were visualized using an ECL detection system (Amersham Imager 600, GE Healthcare, Sweden, or ChemiDoc™ MP, Bio-Rad, USA).

### Plasmid construction and site-directed mutagenesis

The FLAG-tagged human NLRP3 expression plasmid (Origene RC220952) was used as the template for mutagenesis. Point mutations at Arg351 and Arg578 (R351A and R578A) were generated using a site-directed mutagenesis PCR method according to the manufacturer’s instructions (EZchange™ Site-directed Mutagenesis kit, Enzynomics, KOREA). Mutagenic primers were designed to substitute arginine residues with alanine at the indicated positions. PCR-amplified products were treated with DpnI to remove the parental methylated DNA template and transformed into Escherichia coli competent cells. Plasmid DNA was purified using a plasmid purification kit (higene plasmid miniprep kit, BioFACT ), and the presence of the intended mutations was confirmed by Sanger sequencing. The sequences of mutagenic primers used in this study are listed in Supplementary Table 1.

### Co-immunoprecipitation assay

HEK293T cells were co-transfected with plasmids encoding FLAG-tagged NLRP3 (WT), FLAG-tagged NLRP3 mutants (R351A or R578A) (5 µg each), together with HA-tagged NEK7 (5 µg), using Lipofectamine 2000 according to the manufacturer’s instructions. After 36 h of transfection, cells were lysed in TTNE lysis buffer (1% Triton X-100, 150 mM NaCl, 50 mM Tris-HCl, 2 mM EDTA, pH 7.5), and protein concentrations were determined using a BCA protein assay.

Equal amounts of protein lysates were pre-cleared with Sepharose 4B beads for 2 h at 4 °C to reduce non-specific binding. The lysates were then incubated with Anti-FLAG M2 affinity gel for 4 h at 4 °C with gentle rotation to immunoprecipitate FLAG-tagged NLRP3. The beads were washed five times with lysis buffer and subsequently mixed with 2× SDS sample buffer and boiled for 5 min. The immunoprecipitated proteins were analyzed by SDS–PAGE followed by Western blotting.

### SDD-AGE for NLRP3 oligomerization

NLRP3 oligomerization was analyzed by semi-denaturing detergent agarose gel electrophoresis (SDD-AGE). Briefly, bone marrow-derived macrophages (BMDMs) were primed with lipopolysaccharide (LPS, 100 ng/ml) and pretreated with DPD for 1 h, followed by stimulation with nigericin (10 µM) for 30 min to activate the NLRP3 inflammasome. After stimulation, culture supernatants were removed, and cells were washed twice with ice-cold PBS. Cells were lysed in TTNE buffer (1% Triton X-100, 150 mM NaCl, 50 mM Tris-HCl, 2 mM EDTA, pH 7.5) containing protease inhibitors on ice for 20 min. Lysates were centrifuged at 500 × g for 10 min at 4 °C to separate soluble and insoluble fractions.

The soluble fractions (supernatants) were collected for protein quantification and normalization, while the pellets were washed twice with PBS by centrifugation at 500 × g for 10 min. After washing, pellets were resuspended in 1× sample buffer (0.5× TBE, 10% glycerol, 2% SDS, and 0.0025% bromophenol blue). Insoluble fractions were resolved on a 1.5% vertical agarose gel under semi-denaturing conditions using 1× TBE running buffer containing 0.1% SDS at 100 V for 1 h. Proteins were transferred to PVDF membranes using transfer buffer (10% methanol, 0.05% SDS) at 30 V for 800 min on ice. Membranes were blocked with 10% skim milk and probed with anti-NLRP3/NALP3 (Cryo-2) antibody (AG-20B-0014-C100, AdipoGen) to detect oligomerized NLRP3.

### ASC oligomerization assay

BMDMs were lysed in A0 buffer (0.5% Triton X-100, 20 mM HEPES-KOH, 150 mM KCl, pH 7.5) containing protease inhibitors for 20 min on ice. Lysates were centrifuged (6000 rpm, 4 °C, 15 min), washed with A0 buffer and PBS, and resuspended in PBS. Cross-linking was performed with DSS (2 mM) for 30 min at room temperature, followed by quenching with 20 mM Tris-HCl (pH 7.5). Pellets were centrifuged (13,000 rpm, 15 min) and dissolved in SDS sample buffer for immunoblotting.

### Fractionation of soluble and insoluble proteins

Cells were lysed in TTNE buffer (1% Triton X-100, 150 mM NaCl, 50 mM Tris, 2 mM EDTA, pH 7.5) containing protease inhibitors for 20 min on ice and centrifuged at 13,000 rpm for 15 min. The supernatant (soluble fraction) and pellet (insoluble fraction) were separated and analyzed by immunoblotting.

### Immunocytochemistry

For ASC speck staining, BMDMs (3 × 10^5^ cells/well) were seeded on poly-L-lysine-coated coverslips in 12-well plates (SPL, 30012, Korea). After inflammasome stimulation, cells were fixed with 4% paraformaldehyde for 15 min and blocked with 10% horse serum in 1% Triton X-100/PBS for 1 h. Cells were incubated overnight at 4 °C with anti-ASC antibody (1:200), followed by Cy3-conjugated goat anti-mouse secondary antibody (1:300) for 1 h at room temperature in the dark. Nuclei were counterstained with DAPI (30 ng/ml), and slides were mounted with antifade reagent. Fluorescence images were obtained using a fluorescence microscope (ECLIPSE Ts2R, Nikon, Japan).

### Drug affinity responsive target stability (DARTS) assay

LPS-primed BMDMs were lysed in TTNE buffer containing protease inhibitors at 4 °C for 20 min and centrifuged (13,000 rpm, 15 min). Equal protein amounts were incubated with DMSO or the indicated concentrations of DPD for 30 min, followed by limited proteolysis. Samples were analyzed by immunoblotting to assess the interaction of DPD with NLRP3 and NEK7^30^.

### In vivo experiments

LPS (20 mg/kg) was injected intraperitoneally to mice to induce septic shock. DPD in two different doses (12.5 mg/kg and 25 mg/kg) and MCC950 (20 mg/kg) was injected 2 h prior of LPS injection. After 3 h the mouse serum was collected to measure cytokine levels.

DPD in two different doses (12.5 mg/kg and 25 mg/kg) and MCC950 (20 mg/kg) was administered intraperitoneally (i.p) injection before 1 h min i.p injection of MSU crystals (50 mg/kg). After 6 h, mice were sacrificed and performed peritoneal washing. The peritoneal lavage fluid was collected and centrifuged. The supernatant was assayed by ELISA method. All mice were euthanized by CO_2_ inhalation using a regulated CO_2_ flow system, and followed by cervical dislocation to ensure death.

### In silico docking

The crystal structure of human NLRP3-NACHT domain (PDB:7ALV, 2.8 Ǻ) was obtained from the Protein Data Bank. Water molecules and an inhibitor were removed from this structure. Then protein structure was energy-minimized in Yasara energy minimization server. Structure of DPD was prepared and optimized in Gaussian 09 software. The structure of MCC950 was downloaded from PubChem. Molecular docking study was performed using Autodock Vina software and the results were analyzed in Discovery Studio software.

### Statistical analysis

All data are presented as mean ± standard error of the mean (SEM) from at least three independent experiments. Statistical analyses were performed using GraphPad Prism 5. Differences between groups were analyzed using one-way analysis of variance (ANOVA) followed by Tukey’s post hoc test unless otherwise specified. *P* < 0.05 was considered statistically significant. The number of biological replicates (n) and the specific statistical tests used are indicated in the figure legends.

## Supplementary Information

Below is the link to the electronic supplementary material.

Supplementary Material 1

Supplementary Material 2

## Data Availability

The datasets generated and/or analyzed during the current study are available from the corresponding author on reasonable request. The datasets generated during this study do not include high-throughput or novel sequencing data. Sanger sequencing was performed solely to confirm plasmid mutations, and thus data deposition in a public repository is not applicable. All relevant information is provided within the article its supplementary materials.

## References

[1] Latz, E., Xiao, T. S. & Stutz, A. Activation and regulation of the inflammasomes. *Nat. Rev. Immunol.* **13**, 397 (2013). 23702978 10.1038/nri3452 PMC3807999

[2] Broz, P. & Dixit, V. M. Inflammasomes: Mechanism of assembly, regulation and signalling. *Nat. Rev. Immunol.* **16**, 407 (2016). 27291964 10.1038/nri.2016.58

[3] Martinon, F., Pétrilli, V., Mayor, A., Tardivel, A. & Tschopp, J. Gout-associated uric acid crystals activate the NALP3 inflammasome. *Nature* **440**, 237 (2006). 16407889 10.1038/nature04516

[4] Wen, H. et al. Fatty acid-induced NLRP3-ASC inflammasome activation interferes with insulin signaling. *Nat. Immunol.* **12**, 408 (2011). 21478880 10.1038/ni.2022 PMC4090391

[5] Yan, Y. et al. Dopamine controls systemic inflammation through inhibition of NLRP3 inflammasome. *Cell* **160**, 62 (2015). 25594175 10.1016/j.cell.2014.11.047

[6] Monnerat, G. et al. Macrophage-dependent IL-1β production induces cardiac arrhythmias in diabetic mice. *Nat. Commun.* **7** (2016). 10.1038/ncomms13344 PMC5123037 27882934

[7] Panicker, N. et al. Neuronal NLRP3 is a parkin substrate that drives neurodegeneration in Parkinson’s disease. *Neuron* **110**, 2422 (2022). 35654037 10.1016/j.neuron.2022.05.009 PMC9357148

[8] Yamasaki, K. et al. NLRP3/cryopyrin is necessary for interleukin-1β (IL-1β) release in response to hyaluronan, an endogenous trigger of inflammation in response to injury. *J. Biol. Chem.* **284**, 12762 (2009). 19258328 10.1074/jbc.M806084200 PMC2676006

[9] Guo, H., Callaway, J. B. & Ting, N. Y. Inflammasomes: Mechanism of action, role in disease, and therapeutics. *Nat. Med.* **21**, 677 (2015). 26121197 10.1038/nm.3893 PMC4519035

[10] Jiang, H. et al. Identification of a selective and direct NLRP3 inhibitor to treat inflammatory disorders. *J. Exp. Med.* **214**, 3219 (2017). 29021150 10.1084/jem.20171419 PMC5679172

[11] Coll, R. C. et al. MCC950 directly targets the NLRP3 ATP-hydrolysis motif for inflammasome inhibition. *Nat. Chem. Biol.* **15**, 556 (2019). 31086327 10.1038/s41589-019-0277-7

[12] Marchetti, C. et al. OLT1177, a β-sulfonyl nitrile compound, safe in humans, inhibits the NLRP3 inflammasome and reverses the metabolic cost of inflammation. *Proc. Natl. Acad. Sci. U. S. A.* **115** (2018). 10.1073/pnas.1716095115 PMC5816172 29378952

[13] Mangan, M. S. J. et al. Targeting the NLRP3 inflammasome in inflammatory diseases. *Nat. Rev. Drug Discov*. **17**, 588 (2018). 30026524 10.1038/nrd.2018.97

[14] Darakhshan, S. & Pour, A. B. Tranilast: A review of its therapeutic applications. *Pharmacol. Res.* **91**, 15 (2014). 25447595 10.1016/j.phrs.2014.10.009

[15] Swanson, K. V., Deng, M. & Ting, N. Y. The NLRP3 inflammasome: Molecular activation and regulation to therapeutics. *Nat. Rev. Immunol.* **19**, 477 (2021). 10.1038/s41577-019-0165-0 PMC7807242 31036962

[16] Kang, J. et al. Novel activity of ODZ10117, a STAT3 inhibitor, for regulation of NLRP3 inflammasome activation. *IJMS*. **24** (2023). 10.3390/ijms24076079 PMC10094431 37047051

[17] Hornung, V. et al. AIM2 recognizes cytosolic dsDNA and forms a caspase-1-activating inflammasome with ASC. *Nature*. **458**, 514 (2009). 19158675 10.1038/nature07725 PMC2726264

[18] Zhao, Y. et al. The NLRC4 inflammasome receptors for bacterial flagellin and type III secretion apparatus. *Nature* **477**, 596–600 (2011). 21918512 10.1038/nature10510

[19] Orlowski, G. M. et al. Frontline science: Multiple cathepsins promote inflammasome-independent, particle-induced cell death during NLRP3-dependent IL-1beta activation. *J. Leukoc. Biol.* **102**, 7–17 (2017). 28087651 10.1189/jlb.3HI0316-152R PMC6608057

[20] Hornung, V. et al. Silica crystals and aluminum salts activate the NALP3 inflammasome through phagosomal destabilization. *Nat. Immunol.* **9**, 847–856 (2008). 18604214 10.1038/ni.1631 PMC2834784

[21] Aizawa, E. et al. GSDME-dependent incomplete pyroptosis permits selective IL-1alpha release under caspase-1 inhibition. *iScience*. **23**, 101070 (2020). 32361594 10.1016/j.isci.2020.101070 PMC7200307

[22] Mariathasan, S. et al. Differential activation of the inflammasome by caspase-1 adaptors ASC and Ipaf. *Nature* **430**, 213–218 (2004). 15190255 10.1038/nature02664

[23] Boucher, D. et al. Caspase-1 self-cleavage is an intrinsic mechanism to terminate inflammasome activity. *J. Exp. Med.* **215**, 827–840 (2018). 29432122 10.1084/jem.20172222 PMC5839769

[24] He, W. et al. Gasdermin D is an executor of pyroptosis and required for interleukin-1β secretion. *Cell. Res.* **25**, 1285–1298 (2015). 26611636 10.1038/cr.2015.139 PMC4670995

[25] Schmidt, F. I. et al. A single domain antibody fragment that recognizes the adaptor ASC defines the role of ASC domains in inflammasome assembly. *J. Exp. Med.* **213**, 771 (2016). 27069117 10.1084/jem.20151790 PMC4854733

[26] Lu, A. et al. Unified polymerization mechanism for the assembly of ASC-dependent inflammasomes. *Cell* **156**, 1193–1206 (2014). 24630722 10.1016/j.cell.2014.02.008 PMC4000066

[27] Gross, C. J. et al. K(+) efflux-independent NLRP3 inflammasome activation by small molecules targeting mitochondria. *Immunity* **45**, 761–773 (2016). 27692612 10.1016/j.immuni.2016.08.010

[28] He, Y., Zeng, M. Y., Yang, D., Motro, B. & Nunez, G. NEK7 is an essential mediator of NLRP3 activation downstream of potassium efflux. *Nature* **530**, 354–357 (2016). 26814970 10.1038/nature16959 PMC4810788

[29] Shi, H. NLRP3 activation and mitosis are mutually exclusive events coordinated by NEK7, a new inflammasome component. *Nat. Immunol.* (2015). 10.1038/ni.3333 PMC4862588 26642356

[30] Pai, M. Y. et al. Drug affinity responsive target stability (DARTS) for small-molecule target identification. *Methods Mol. Biol.* **287** (2016). 10.1007/978-1-4939-2269-7_22 PMC4442491 25618353

[31] Patil, S. M. et al. Computational screening of benzophenone integrated derivatives (BIDs) targeting the NACHT domain of the potential target NLRP3 inflammasome. *Adv. Cancer Biol. Metastasis* **5** (2022).

[32] Fu, J. & Wu, H. Structural mechanisms of NLRP3 inflammasome assembly and activation. (2023). 10.1146/annurev-immunol-081022-021207 PMC10159982 36750315

[33] Xiao, L., Magupalli, V. G. & Wu, H. Cryo-EM structures of the active NLRP3 inflammasome disc. *Nature* **613**, 595 (2024). 10.1038/s41586-022-05570-8 PMC10091861 36442502

[34] Hayat, C. et al. Identification of new potent NLRP3 inhibitors by multi-level in-silico approaches. *BMC Chem.* **18** (2024). 10.1186/s13065-024-01178-3 PMC11027297 38637900

[35] Liu, D. & Huang, Y. Protocol for in vivo and in vitro activation of NLRP3 inflammasome in mice using monosodium urate. *STAR Protoc.* **4** (2023). 10.1016/j.xpro.2023.102554 PMC10493585 37682717

[36] Kannan, S. K. et al. Mouse models of sepsis. *Curr. Protoc.* **4** (2024). 10.1002/cpz1.997 PMC10917121 38439603

[37] Kayagaki, N. et al. Caspase-11 cleaves gasdermin D for non-canonical inflammasome signalling. *Nature* **526**, 666–671 (2015). 26375259 10.1038/nature15541

[38] Ruhl, S. & Broz, P. Caspase-11 activates a canonical NLRP3 inflammasome by promoting K(+) efflux. *Eur. J. Immunol.* **45**, 2927–2936 (2015). 26173909 10.1002/eji.201545772

[39] Downs, K. P., Nguyen, H., Dorfleutner, A. & Stehlik, C. An overview of the non-canonical inflammasome. *Mol. Aspects Med.* **76**, 100924 (2020). 33187725 10.1016/j.mam.2020.100924 PMC7808250

[40] El-sayed, S., Freeman, S. & Bryce, R. A. Probing the effect of NEK7 and cofactor interactions on dynamics of NLRP3 monomer using molecular simulation. *Protein Sci.* **31** (2022). 10.1002/pro.4420 PMC9601872 36173167

[41] Ma, Q. Pharmacological inhibition of the NLRP3 inflammasome: Structure, molecular activation, and inhibitor-NLRP3 interaction. *Pharmacol. Rev.* **75**, 487 (2023). 36669831 10.1124/pharmrev.122.000629 PMC10121800

