# Supplementary material for: A derivative of PD156707 selectively inhibits NLRP3 inflammasome activation by directly binding to NLRP3
Source: Sci Rep. 2026 Apr 21;16:18577. doi: 10.1038/s41598-026-49619-4 (PMC13269700; doi:10.1038/s41598-026-49619-4)
Supplement: Supplementary file 1 — Supplementary Material 1 [file 41598_2026_49619_MOESM1_ESM.docx]

Supplementary Information

**A derivative of PD156707 selectively inhibits NLRP3 inflammasome activation by directly binding to NLRP3**

Ye-Rin Jung^1,4^, Xiang Fei^3,4^, Hyeong-Min Lee^1^, Anamul Hasan^1^, Eun-Ji Kim^1^, Kyu Tae Byun^2^, Seung-Yong Seo^3^, Tae-Bong Kang^1,2, *^ and Kwang-Ho Lee^1.^

^1^ Department of Applied Life Science, BK21 Program, Graduate School, Konkuk University, Chungju 27478, Republic of Korea.

^2^ Department of Biotechnology, Research Institute (RIBHS), College of Biomedical and Health Science, Konkuk University, Chungju 27478, Republic of Korea.

^3^ College of Pharmacy, Gachon University, Incheon 21936, Republic of Korea.

^4^ Ye-Rin Jung and Xiang Fei contributed equally to this work

*Present address*: Kwang-Ho. Lee, Elimland Co., Ltd., Gyeonggi-do, 12106, Republic of Korea

*Correspondence and requests for materials should be addressed to T-B.K. (kangtbko@kku.ac.kr)

**Supplementary Methods**

**Assessment of ASC Self-Oligomerization in HEK293T Cells**

HEK293T cells were transfected with a pLVEF1a-mASC-FUGW-IRES-puro plasmid using JetPEI transfection reagent (Polyplus-transfection). Indicated amount of DPD was administered at different time points relative to transfection (pre-, co-, or post-treatment) to evaluate its effect on ASC self-oligomerization. After 24 h, cells were fixed, and ASC speck formation was visualized by fluorescence microscopy (ECLIPSE Ts2R, Nikon, Japan).


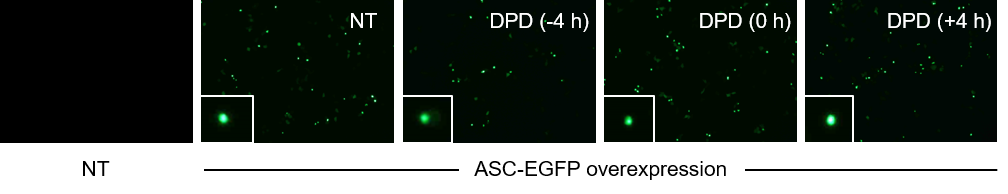


**Supplementary Figure 1**. DPD does not inhibit ASC self-oligomerization induced by ASC overexpression

HEK293T cells were transfected with a plasmid encoding mouse ASC to induce ASC self-oligomerization. DPD was added 4 h prior to transfection (-4 h), at the time of transfection (0 h), or 4 h after transfection (+4 h). After 24 h, ASC speck formation was analyzed by fluorescence microscopy. Robust ASC speck formation was observed in all conditions, and DPD treatment did not reduce ASC oligomerization, indicating that DPD does not directly inhibit ASC self-oligomerization.

**Supplementary Table 1.** Primers used for site-directed mutagenesis of human NLRP3

| Mutation | Primer name | Sequence (5🡪3) |
| --- | --- | --- |
| R351A | NLRP3-R351A-F | CCTGTG**GCC**CTGGAGAAA |
| R351A | NLRP3-R351A-R | GCCGTGGTGATGAGCAGAGA |
| R578A | NLRP3-R578A-F | CTTCCTCTTTG**GCC**TGGTA |
| R578A | NLRP3-R578A-R | GCTACAACAAAAATCAAATAC |
