# Supplementary material for: A derivative of PD156707 selectively inhibits NLRP3 inflammasome activation by directly binding to NLRP3
Source: Sci Rep. 2026 Apr 21;16:18577. doi: 10.1038/s41598-026-49619-4 (PMC13269700; doi:10.1038/s41598-026-49619-4)

Supplementary Fig. S1

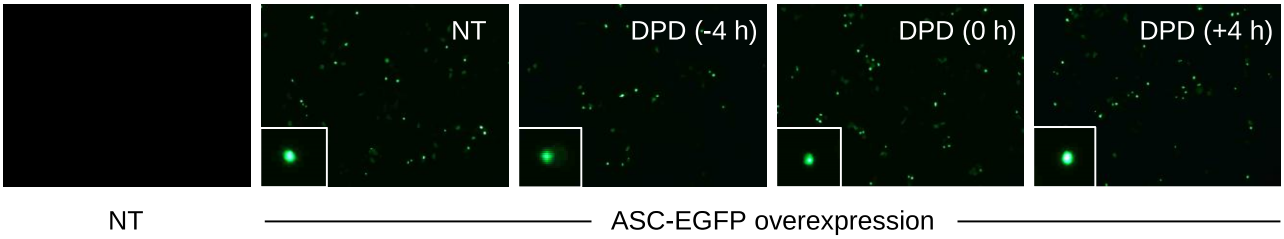

Supplementary Fig. S2 (Full-length blot for Fig. 2e)

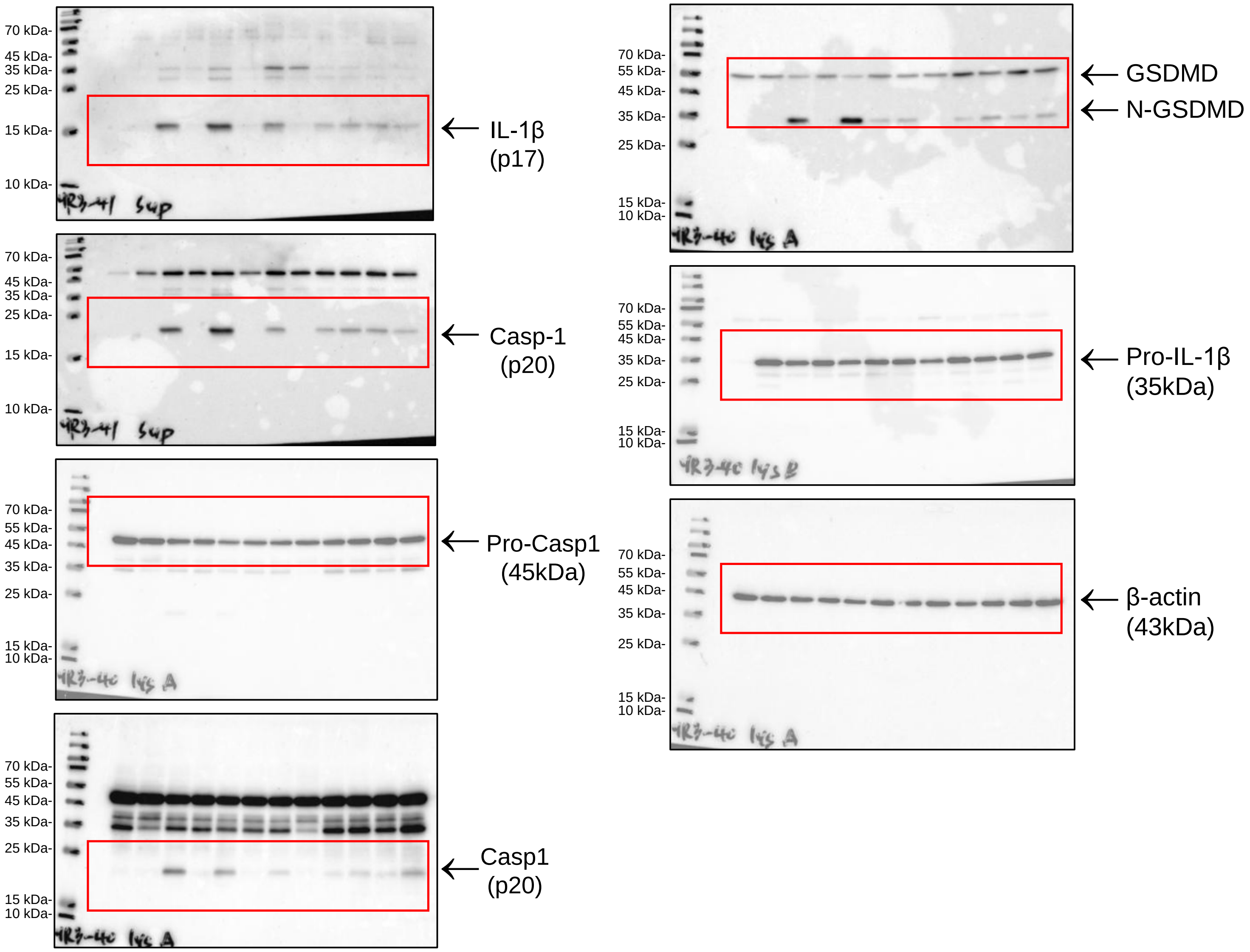

Supplementary Fig. S3 (Full-length blot for Fig. 3a, 3b)

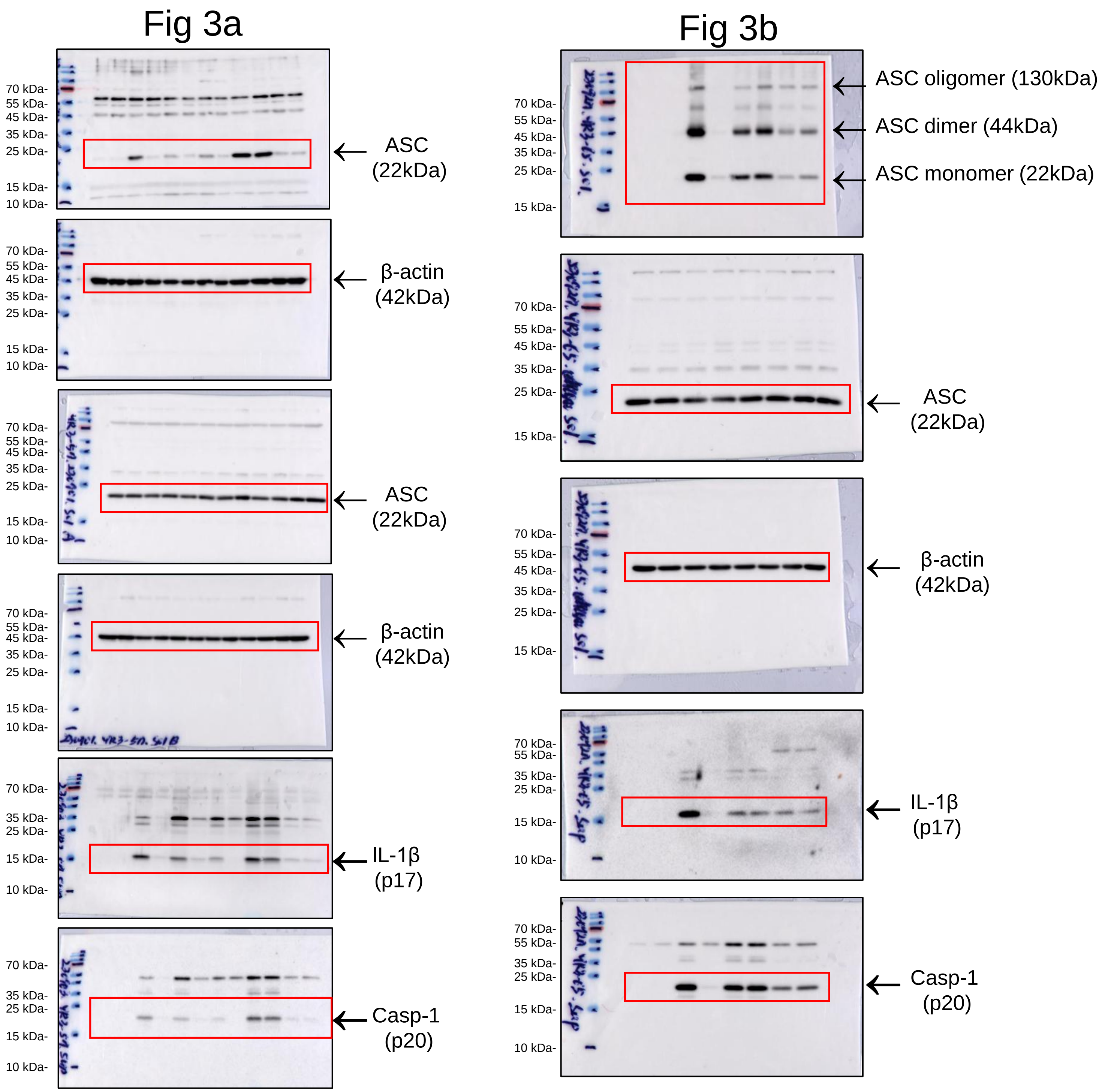

Supplementary Fig. S4 (Full-length blot for Fig. 4a, 4b, 4d)

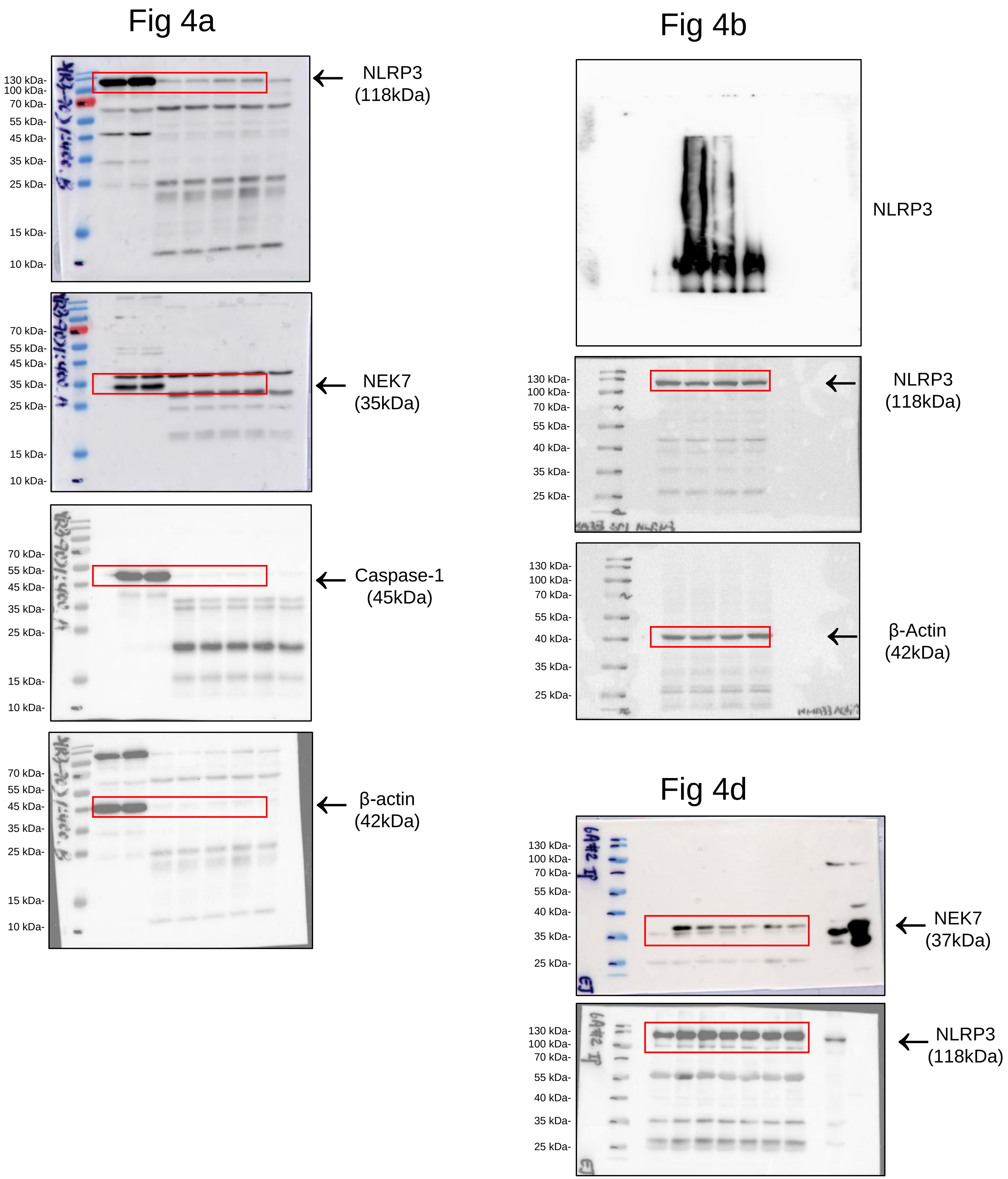

Supplement: Supplementary file 2 — Supplementary Material 2 [file 41598_2026_49619_MOESM2_ESM.pdf]
